# Piezo/sono-catalytic activity of ZnO micro/nanoparticles for ROS generation as function of ultrasound frequencies and dissolved gases

**DOI:** 10.1016/j.ultsonch.2023.106470

**Published:** 2023-06-08

**Authors:** A. Troia, S. Galati, V. Vighetto, V. Cauda

**Affiliations:** a Ultrasounds and Chemistry Lab, Advanced Metrology for Quality of Life, Istituto Nazionale di Ricerca Metrologica, Turin, Italy; b Department of Applied Science and Technology, Polytechnic of Turin, Italy

**Keywords:** Piezocatalytic, Sonocatalytic, ZnO, Ultrasound, Cavitation, OH radicals

## Abstract

We report an accurate study on sonocatalytic properties of different ZnO micro and nanoparticles to enhance OH radical production activated by cavitation. In order to investigate some of the still unsolved aspects related to the piezocatalytic effect, the degradation of Methylene Blue and quantification of radicals production have been evaluated as function of different ultrasonic frequencies (20 kHz and 858 kHz) and dissolved gases (Ar, N_2_ and air). The results shown that at low frequency the catalytic effect of ZnO particles is well evident and influenced by particle dimension while at high frequency a reduction of the degradation efficiency have been observed using larger particles. An increase of radical production have been observed for all ZnO particles tested while the different saturating gases have poor influence. In both ultrasonic set-up the ZnO nanoparticles resulted the most efficient on MB degradation revealing that the enhanced radical production may arise more from bubbles collapse on particles surface than the discharge mechanism activate by mechanical stress on piezoelectric particles. An interpretation of these effects and a possible mechanism which rules the sonocatalytic activity of ZnO will be proposed and discussed.

## Highlights

• Radicals formation as a function of different ZnO nano and micro particles.
• ZnO sonocatalytic activity as function of ultrasonic frequency and dissolved gases
• Identification of relationship between sonocatalytic effect and particle dimensions.
• Investigation of mechanical effects and particles erosion of ZnO particles.

## Graphical abstract

**Figure:**
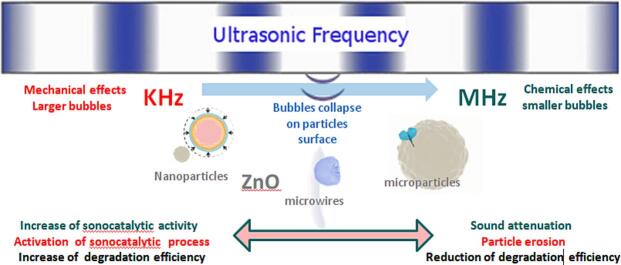

## 1 Introduction

Piezo/Sono-catalysis has emerged in these last years as a tool for enhancing the radicals production by ultrasonic irradiation of aqueous solutions. Despite the generation of radicals in water exposed to high intensity ultrasound is well known, especially as function of ultrasonic frequencies or dissolved gases [1], [2] the mechanism underpinning the enhanced radicals production in presence of micro-nano piezoelectric particles is still unclear despite the high number of publications have been recently reported for environmental [3], [4], [5] biomedical [6] and energetic applications [7], [8], [9].The basic idea is that the presence of piezoelectric particles exposed to mechanical stress given by ultrasonic irradiation can generate radicals from discharge surface effect of the particles. However, the theory describing this effect involves the presence of very high mechanical stresses that should arise from shock waves, generated by bubbles collapse at the particles surface. In this mechanism the particles size and shape seem also to play a role, which is however not yet clarified by the already-reported experimental works [10]. A lot of piezo electric materials have been tested over these last 10 years as barium titanate (BaTiO_3_), titania (TiO_2_), zinc oxide (ZnO), polyvinylidenedifluoride (PVDF), polytetrafluoroethylene (PTFE) and several composites; an exhaustive list can be found here [11]. However most of the studies report on the use of low frequency ultrasound, in particular ultrasonic bath reactors, with different experimental conditions (i.e. various temperatures of the solution, volumes and materials of the vials in case of indirect US transmission, different sample preparations for the analysis) or different monitoring technique (UV–vis measurements for degradation of common dyes, chromatographic analysis for the degradation of organic pollutants, EPR radicals measurement or generation of gases for green energy applications) which lead to different interpretation of the results [12], [13].Some works at high frequency (>500 kHz) have been also reported [14], but since the radicals production in these conditions is higher, it is not easy to discriminate the enhancing effect due to the particle presence with respect to ultrasound irradiation of simple water. Although it is already known that bubble collapses are less violent at higher frequencies, it would not seem possible to figure out the presence of a piezo-catalytic mechanism based on the shock waves caused by bubbles collapse or by mechanical shear stress acting on the particles. In some works, this effect was detected as a function of different ultrasound intensities, since the presence of the particles reduce the cavitations threshold [15]. Another hypothesis involves the possible role of sonoluminescence [16] (SL), since most of the tested particles behave also photocatalytic properties, but the mechanism still remains unclear and the literature data are poor. In view of the above-mentioned unclarities and uncertainties, here we report an accurate study on the piezo-sono catalytic effect of different ZnO nanoparticles and microparticles on the degradation of Methylene Blue (MB) aqueous solutions exposed to different ultrasonic frequencies and as a function of different of dissolved gases. We investigated different ZnO sizes and morphologies, i.e. rod-like shape nanoparticles, 1D microwires and multipods-like microparticles. UV–vis absorption and Electron Paramagnetic Resonance (EPR) measurements have revealed a clear effect of ZnO particles to promote the MB degradation and enhance the radicals production using low frequency sonotrode (20 kHz) reactor. On the other side the experiments performed with high frequency (858 kHz) ultrasonic bath revealed that this enhancing effect is less evident because of the amount of radicals produced by high ultrasound frequencies even without ZnO particles. In general, nanostructured ZnO seems to be more effective than microparticles, which may appear in contrast to what is reported in extensive review papers [12], [13]in which larger particles with dimension of tens or hundreds of micron should be ideal for activating piezo-catalytic processes. This discrepancy confirms the need of further investigations about the effect of the particles size, especially in the range of nano-sized and micro-sized piezoelectric materials, as claimed also in [17].

The results obtained with different gases suggest that the presence of dissolved oxygen in the solution is necessary to activate the mechanism, while Ar saturation of ZnO solutions accelerates the rate of MB degradation, and could be correlated to the well-known [18] increase of OH· radicals formation in presence of noble gas. FESEM analysis on ZnO particles after ultrasonic treatments evidenced a dramatic modification of the shape of larger microparticles, while no modifications appeared on nanoparticles and microwires. The obtained results clearly indicate that the synergistic effect of ZnO particles is influenced by ultrasonic frequency, particles dimension, particle shape and the dissolved gas. An interpretation of the role of these parameters on the activation of piezo/sonocatalityc activity is proposed and discussed.

## 2 Materials and methods

### 2.1 Ultrasonic reactors

**Fig. 1:**
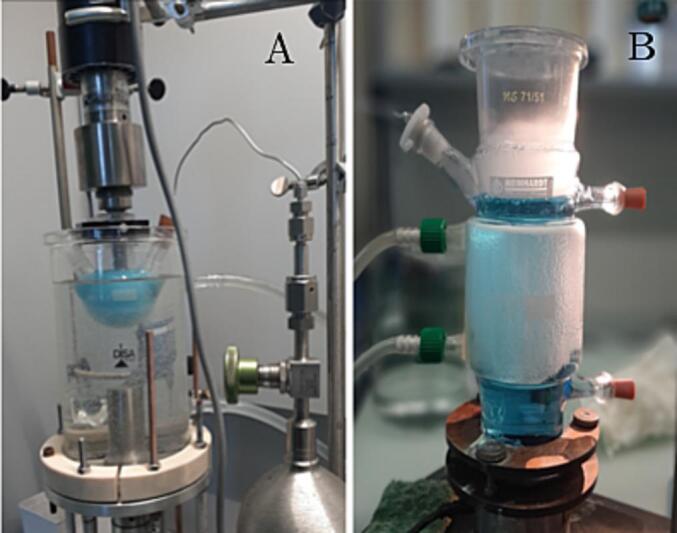
Overview of the two experimental apparatus used for ZnO sonocatalytic degradation of MB in presence of different gas at 20 kHz (A) and 858 kHz (B).

Two ultrasonic reactors set-up have been used in our experiments. One consist of a 20 kHz sonotrode (Bandelin HD2200) with 13 mm Titanium tip (TT13) immersed into the solution (50 ml glass flask) and kept at constant ambient temperature by means of water cooling bath (picture A of Fig. 1). Second apparatus consist of a 858 kHz transducer driven by function generator (Agilent 33250A) and amplifier (MEINHARDT Ultrasonics E/805/T), equipped with a 400 ml glass reactor and kept at ambient temperature by means of cooling jacket connected to a thermostatic bath (picture B of Fig. 1).Ultrasonic power has been kept constant during all the treatments for each set-up. Briefly, 20 kHz sonotrode worked at 20% of the maximum power which correspond to our previous calorimetric methods [19] to 2.5 W, while for 858 kHz transducer the acoustic field was characterize by means of needle hydrophone (Müller-Platte Needle Probe) and a mean pressure of 1.3 MPa have been used for our experiments. Ultrasonic treatments have been performed in dissolved air, or under Ar or N_2_ saturation for a time of 45, 60 and 90 min. EPR measurements required smaller amounts of solution, thus small glass vials filled with 2 or 4 ml solution containing ZnO catalysts (0.1% in weight) have been immersed in a thermostatic bath and treated indirectly with the high frequency transducer operating at same acoustic pressure, while a small titanium exponential probe (MS73, 2 mm of diameter) operating at 10% of maximum power and immersed into the vials has been used for low frequency experiments.

### 2.2 Samples preparation

**Fig. 2:**
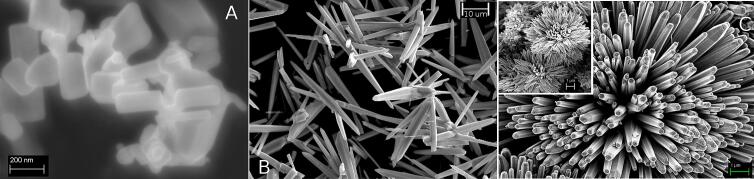
Microscopic (FESEM) images of the different ZnO nanorods and microparticles used for these experiments (picture A marker 200 nm, picture B marker 10 μm, picture C markers 1 μm).

Three different types of ZnO particles have been used. The whole preparation details are reported in [20], [21]. Briefly ZnO nanoparticles (ZnO-np) in particular nanorods shaped with dimension ranging between 50 and 100 nm, ZnO microwires (ZNO-mw) with length of 10 um and thickness of 100 nm and ZnO microparticles called “multipods” (ZnO-mps) with round shape (with average diameter of 10 um covered by needles like surface with dimension similar to microwires (see Fig. 2 for electron microscopy overview of the used particles). All the particles have been dispersed at concentration of 0,1 % in weight using a batch solutions (0.025 mM) of Methylene Blue kept under dark conditions as in the case for ultrasonic treatments. After each experiment, samples have been collected and centrifugated (speed 4000 rpm for 5 min) and the supernatant was analyzed by UV–vis absorption.

### 2.3 Instrumental equipments

To evaluate the degradation efficiency of ZnO particles UV–vis absorbance measurements have been performed using a Nicolet GENESYS 50 UV–vis spectrometer by calculating of the total area of absorbance peak of MB spectra in the visible region (from 520 to 720 nm, see SI file for raw examples data) with respect to relative starting solution. All the experiments have been repeated three times and pure water has been used as blank. EPR investigations have been performed by EPR Spectroscopy using a EMXNanoX-Band spectrometer from Bruker assisted by a spin-trapping technique (dimethylpirrolidone – DMPO, Sigma at10 mM. Spectra were recorded with the following measurement conditions: center field 3428 G, sweep time 160.0 s, sample g-factor 2.00000, number of scans 10. The analysis of the recorded spectra was executed using the Bruker SpinFit software. Particles size distribution after ultrasonic treatments have been performed through dynamic light scattering (DLS) using a Delsanano-C from Beckman Coulter. Finally, scanning electron microscopyinvestigations on sonicated particles have been performed with a FEI Inspect-F FESEM.

## 3 Experimental results and discussion

### 3.1 Degradation of MB at 20 kHz

**Fig. 3:**
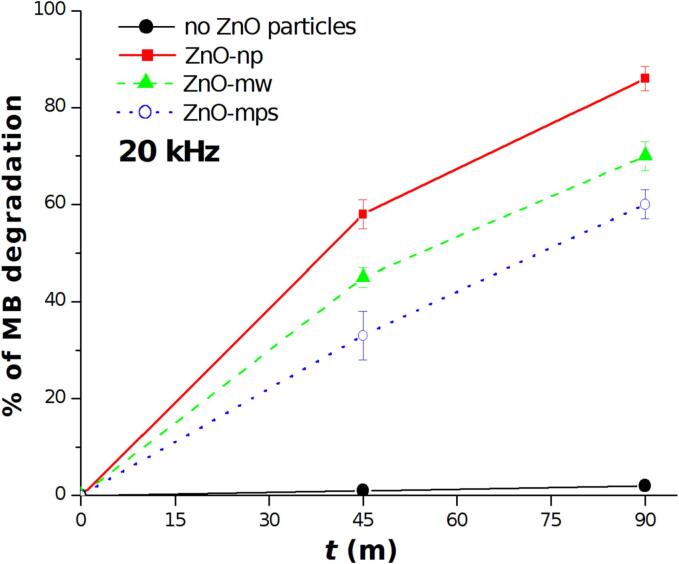
Degradation of MB at 20 kHz in presence of ZnO nanoparticles (red line) and microparticles (1D microwires – green line and multipods – blue line) and in pure aqueous solution (black line).

In Fig. 3 is shown the MB degradation efficiency at 20 kHz as a function of the different ZnO particles for treatment times of 45 and 90 min. The degradation of MBusing only ultrasonic irradiation for 90 min is also reported for comparison (dark line). The effect of ZnO particles is clearly evident: more than 85% of degradation have been obtained using ZnO nanoparticles which resulted the most efficient treatment (red line). With ZnO-mws and ZnO-mps only 70 and60 % of degradation have been reached, respectively, at the same conditions. The “microwires” appeared slight more efficient than “multipods”.

### 3.2 Degradation of MB at 858 kHz

**Fig. 4:**
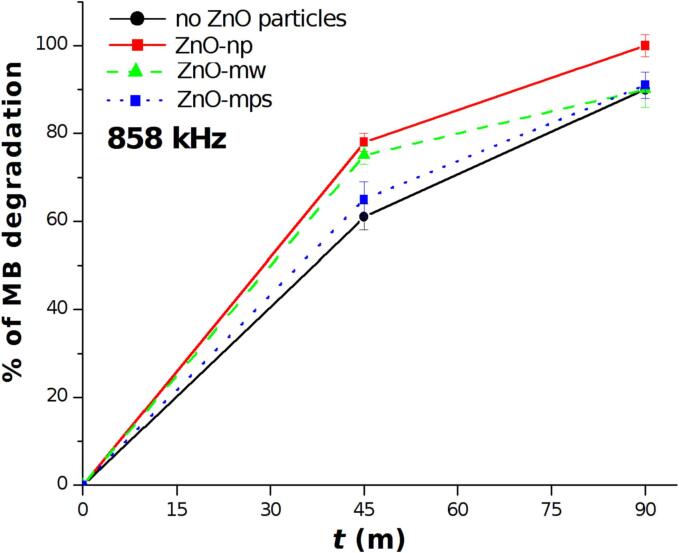
Degradation of MB at 858 kHz in presence of ZnO nanoparticles (red line) and microparticles (1D microwires – green line and multipods – blue line) and in pure aqueous solution (black line).

In Fig. 4 the degradation efficiency at 858 kHz as function of different types of ZnO particles for treatments times of 45 and 90 min is shown. In this case a complete degradation of MB was obtained using the ZnO-np (red line), while using ZnO-mw and ZnO-mps almost 90% of degradation was reached in both cases (green and blue lines), which however is very similar to the degradation obtained using only ultrasonic irradiation for equivalent time (black line).

### 3.3 Effect of different gases

**Fig. 5:**
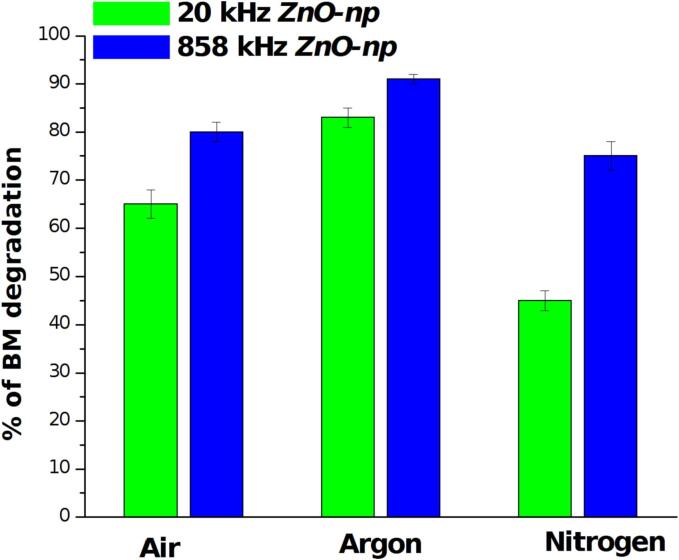
Degradation of MB after 60 min of treatment in presence of ZnO nanoparticles as function of different saturating gases at 20 kHz and 858 kHz.

The role of different saturating gases have been performed through gas bubbling using capillary steel tube during the ultrasound treatment (visible in Fig. 1A). In this case UV–vis measurements have been conducted after 60 min of ultrasonic treatments for each gas (Ar, N_2_ or air) using only ZnO-np. Fig. 5 summarizes the degradation efficiency for both experimental set-up and it is possible to see how in presence of Ar a higher degradation have been achieved with respect to air saturation, while in presence of N_2_ the rate of reaction is slowed down; in particular these effects appeared more evident with low frequency set-up.

### 3.4 EPR measurements

**Fig. 6:**
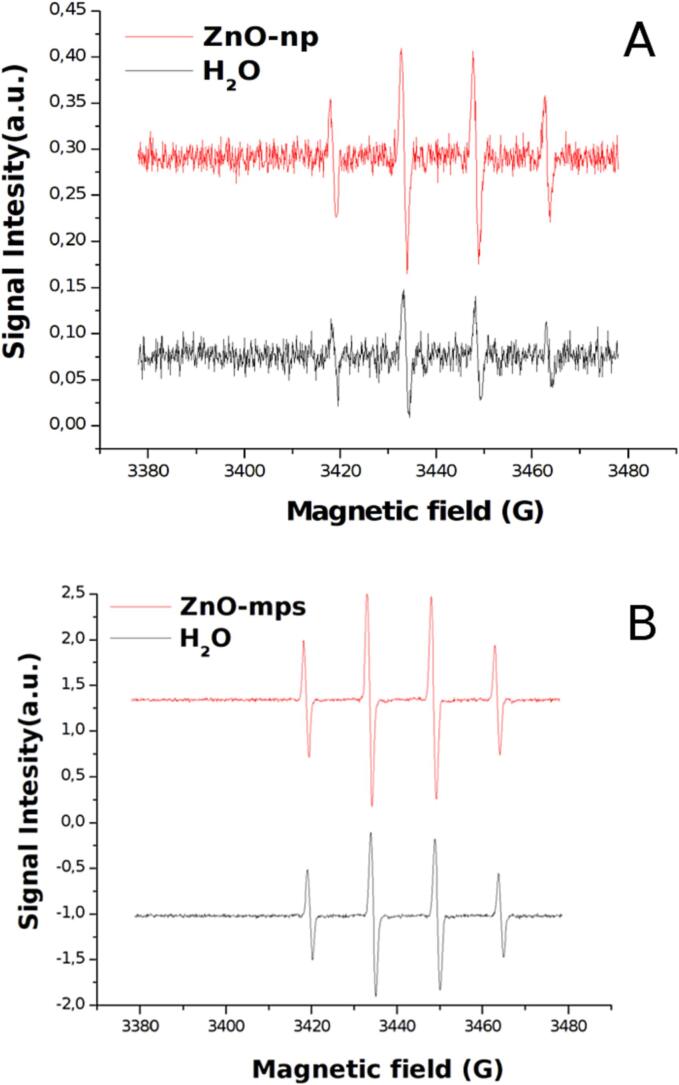
Examples of EPR signals at 20 kHz (A) and 858 kHz (B) in pure water (black line) and in presence of ZnO particles (red line).

**Fig. 7:**
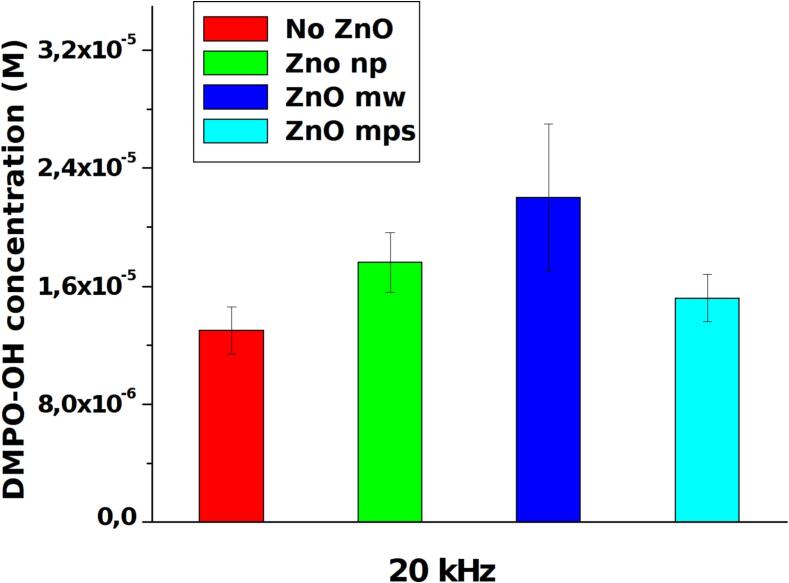
Quantitative evaluation of OH radical produced at 20 kHz in presence of different ZnO particles compared to pure water.

**Fig. 8:**
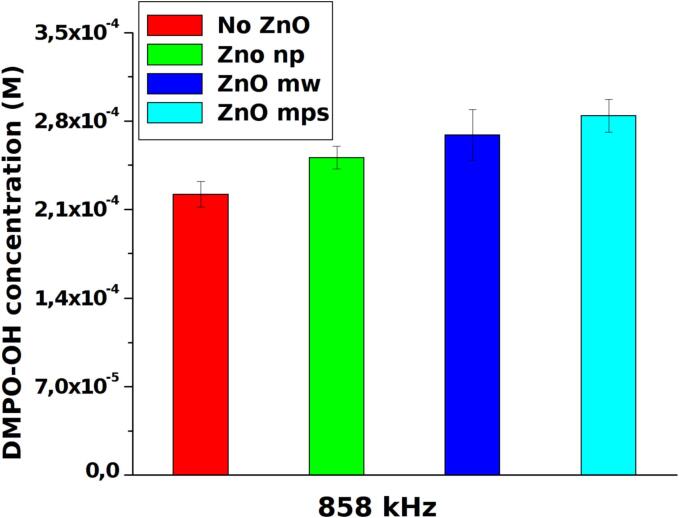
Quantitative evaluation of OH radical produced at 858 kHz in presence of different ZnO particles compared to pure water.

To detect and quantify the production of OH radicals, the EPR measurement was performed in a modified experimental set-up. In order to reduce spin trapping molecules consumption different measurements have been performed on smaller amount of solution (2–4 ml) using the experimental set-up described above for a time of 5 or 10 min depending on volume and DMPO concentrations (0.1–0.2 mM). The temperature of the solution during the treatment was maintained constant by means of a thermostatic bath. DMPO-OH spectra have been collected immediately after ultrasonic irradiation. In Fig. 6A and 6B are shown two examples of EPR raw data respectively at low and high frequency which shows the relative increase of signal in presence of ZnO particles. In Fig. 7, Fig. 8 the quantification of OH radicals measured at 20 kHz and 858 kHz, respectively, as a function of different ZnO particles are reported: each value is the average of three experiments. An increase of OH radicals have been detected in presence of ZnO particles. However, because of high uncertainty, it appears difficult to correlate these results with the UV–vis degradation. A possible reason may originate from other effects occurring during longer treatments, as ultrasound attenuation at high frequency or particles modification at low frequency can be considered, as reported in the discussion section.

### 3.5 DLS and microscopic investigations

**Fig. 9:**
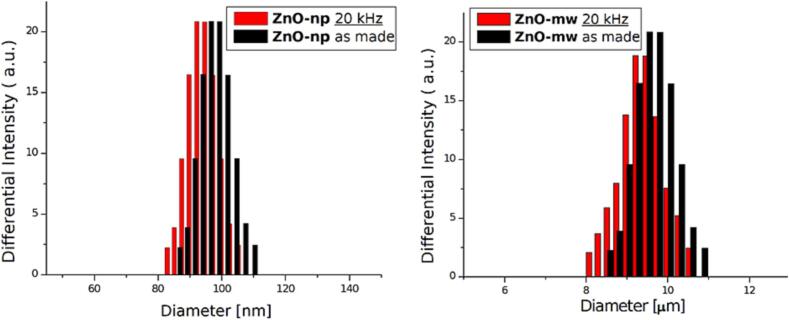
DLS of ZnO nanoparticles (left) and ZnO microwires (right) solutions after 90 min of ultrasonic treatment at 20 kHz compared to as made particles.

**Fig. 10:**
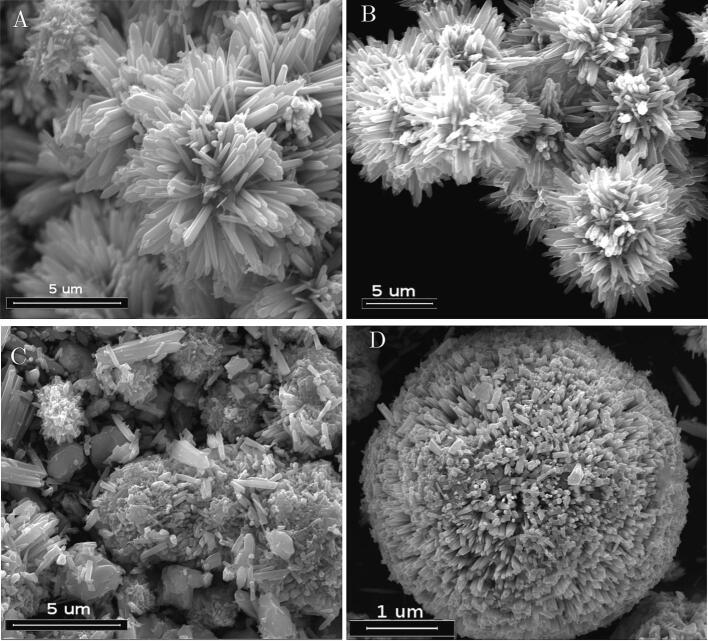
SEM investigations of ZnO-mp (multipods) particles after 90 min of ultrasonic treatment 20 kHz: A and B images of as made particles; C large view of the eroded particles with smaller residuals; D close view of a particles in which the needles composing the structured surface were completely ripped off.

Dynamic light scattering (DLS) measurements have been performed on diluted ZnO samples solutions in water (1:5) since 0.1% was too concentrated and unstable to carry out the measurement. In Fig. 9the population curves for ZnO-np and ZnO-mw after ultrasonic treatment at 20 kHz are shown. It can be clearly noted that both ZnO-np than ZnO-mw remained almost unaltered. The slight difference for ZnO-mw could be due to some breakage of connected microwires structures visible in Fig. 2B prior to sonication experiments. Ultrasonic treatment of ZnO-mps on the contrary caused a great modification of the surface of the particles as revealed by FESEM observations (see Fig. 10). In this case the mechanical action of bubbles collapse gives raise to the breakage of the surface structure of the particles, with formation of smaller particles; DLS on this sample was too unstable to carry out reliable measurements. However, it can be affirmed that at 20 kHz the mechanical effects on this particle type bring to erosion and formation of submicron sized particles. In contrast, no particle modification was observed using the high frequency sonoreactor.

## 4 Discussion

ZnO microparticles and nanoparticles have been widely tested in piezocatalytic experiments since its first report [8], however an investigation on the efficiency of a catalyst as a function of particles dimension, the ultrasound frequencies and the dissolved gas have not been reported before. Recent reviews [22], [23] on this research field stressed out the need of more investigations on these techniques which have been proposed for several applications using a wide type of particles with different shapes and dimensions and by monitoring the efficiency with different ultrasonic experimental set up. The results obtained at high frequency (858 kHz) indicate that the enhancement of degradation efficiency activated by ZnO is mostly negligible with respect to the degradation which normally occurs in pure MB solutions at this frequency. As shown by UV–vis measurements, an increase of degradation efficiency is evident only when using ZnO-np while with ZnO-mw and ZnO-mps the efficiency was almost similar or smaller than the one obtained using simple aqueous solutions. However, EPR revealed an increase of OH radicals in presence of all ZnO particle types. These two findings, which may appear in contrast, are in line with observations reported by other authors [24] which evaluated hydrogen (H_2_) production rate with different metal oxides at 362 kHz. In that case the presence of larger particles of ThO_2_ (∼5 um) slowdown the rate of H_2_ production because of sound attenuation which reduce cavitation activity respect of using pure water or nanosized oxides. Our results at high frequencies confirm these effect using different sized ZnO particles. From one side, the enhancement of bubble nucleation in presence of the solid-state particles leads to the increase of the number of cavitation events, favoring the radicals formation, as it has been found in our previous observations [25]. From the other side, the attenuation of ultrasound waves by larger particles causes a reduction of bubble clouds volume followed by losses of ultrasonic energy released into the reactor and consequent decrease of the sonochemical activity. Using ZnO nanoparticles the attenuation is negligible and the synergistic effect of ZnO allowed to increase the degradation efficiency at high frequency.

The experiments at 20 kHz have evidenced the role of ZnO particles on enhancing the radicals production, since in aqueous solution no degradation of MB occurred even after 90 min of treatment. As shown in Fig. 3, all ZnO particles promote the MB degradation with ZnO-np being the most efficient sonocatalyzer. This result goes in the direction of what have been previously reported [26]: in contrast to what predicted by the models (where larger and more oriented crystals would be preferable to activate the piezocatalytic processes exploiting the mechanical effects, typical at the low frequencies), the authors found that nanoparticles resulted, unexpectedly, to be more efficient. As it was discussed by the same authors in [13] and in another recent review [27], several studies report the use of nanosized catalysts demonstrating that other mechanisms should be taken into account. In another case [28], ZnO nanowires appeared more efficient than ZnO nanoparticles using low intensity ultrasound, revealing as also ultrasonic parameters have to be taken in account indicating that a lot of experimental trials are needed to reach a better comprehension of the phenomenon. In our experiments, an effect of high intensity ultrasound has been shown by microscopic investigations and particles size analysis, thus it is possible assume that the larger the particles, the higher the breakage/erosion. This can cause an energy loss favoring also coalescence phenomena and reducing the efficiency of ZnO-mw and ZnO-mps in the MB degradation. As this process will reasonably took place after several minutes of ultrasonic irradiation,EPR measurements are difficult to correlate since for shorter irradiation times the increase of OH radicals is similar for each ZnO particles tested, despite a relatively high uncertainty. These results suggest that the piezocatalytic process arise from bubbles collapse on the particles surface which activate and promote radicals formations as proposed also in [29]. In this sense the nanoparticles would not affect the mechanism by sound attenuation, particles breakage or other energy absorbing phenomena, but increase the number of active sites for cavitating bubbles making more efficient the degradation activated by the synergistic process. The degradation efficiency as function of different saturating gases in presence of ZnO-np revealed a slight increase in presence of Ar with respect to air and N_2_, in agreement to what have been observed in [30], [31]. In particular at 858 kHz, this effect is less evident, as the reaction mechanism and release of OH• radicals into the bulk is more efficient at higher frequency, even under air saturation. At 20 kHz the effect of noble gas seems more evident. Since it is well known that at low frequency the presence of Ar increase the OH• radicals production in multibubbles sonoluminescence spectra, one would expect an higher degradation efficiency under noble gas saturation. However in presence of ZnO-np, this increase seems to be hidden by the sonocatalytic activity of the particles. The results obtained with N_2_confirm the observation reported in [30] indicating that oxygen participates to the mechanism of OH radicals formation, especially at low frequency. Of course in our experiments these effects have been modified by the catalytic activity of ZnO nanoparticles.

## 5 Conclusion

In this paper we reported an accurate study on ZnO particles with different morphologies for the degradation of Methylene Blue as a function of different ultrasonic frequencies and dissolved gases. The results show a remarkable degradation effect, in particular ZnO nanoparticles at both frequencies. The results obtained with ZnO microparticles revealed that the presence of larger particles affects the phenomenon and reduce the efficiency. This mechanism appears to be poorly influenced by the saturating gas type, despite a slight increase of Ar at 20 kHz. In conclusion, this study reveals that nanosized catalysts are preferable for the investigation of the piezocatalytc processes and should be tested to increase the efficiency and the applications of this technique. Further tests on different particles or by means of other experimental measurement techniques are needed for better understanding of the phenomenon, especially to elucidate its mechanism, which appears a combination of mechanical and chemical activation of piezoelectric particles ruled by bubbles collapse on particles surface.

## Declaration of Competing Interest

The authors declare that they have no known competing financial interests or personal relationships that could have appeared to influence the work reported in this paper.
