# Supplementary material for: Piezo/sono-catalytic activity of ZnO micro/nanoparticles for ROS generation as function of ultrasound frequencies and dissolved gases
Source: Ultrason Sonochem. 2023 Jun 8;97:106470. doi: 10.1016/j.ultsonch.2023.106470 (PMC10311144; doi:10.1016/j.ultsonch.2023.106470)
Supplement: Supplementary data 1 [file mmc1.docx]

Supporting informations of the paper: “*Piezo/sono-catalytic activity of ZnO micro/nanoparticles for ROS generation as function of ultrasound frequencies and dissolved gases”*

*A.Troia^1^, S. Galati^1^, V. Vighetto^2^, V Cauda^2^*

*1 Ultrasounds and Chemistry Lab, Advanced Metrology for Quality of Life, Istituto Nazionale di Ricerca Metrologica, Turin, Italy. 2 Department of Applied Science and Technology, Polytechnic of Turin, Italy.*


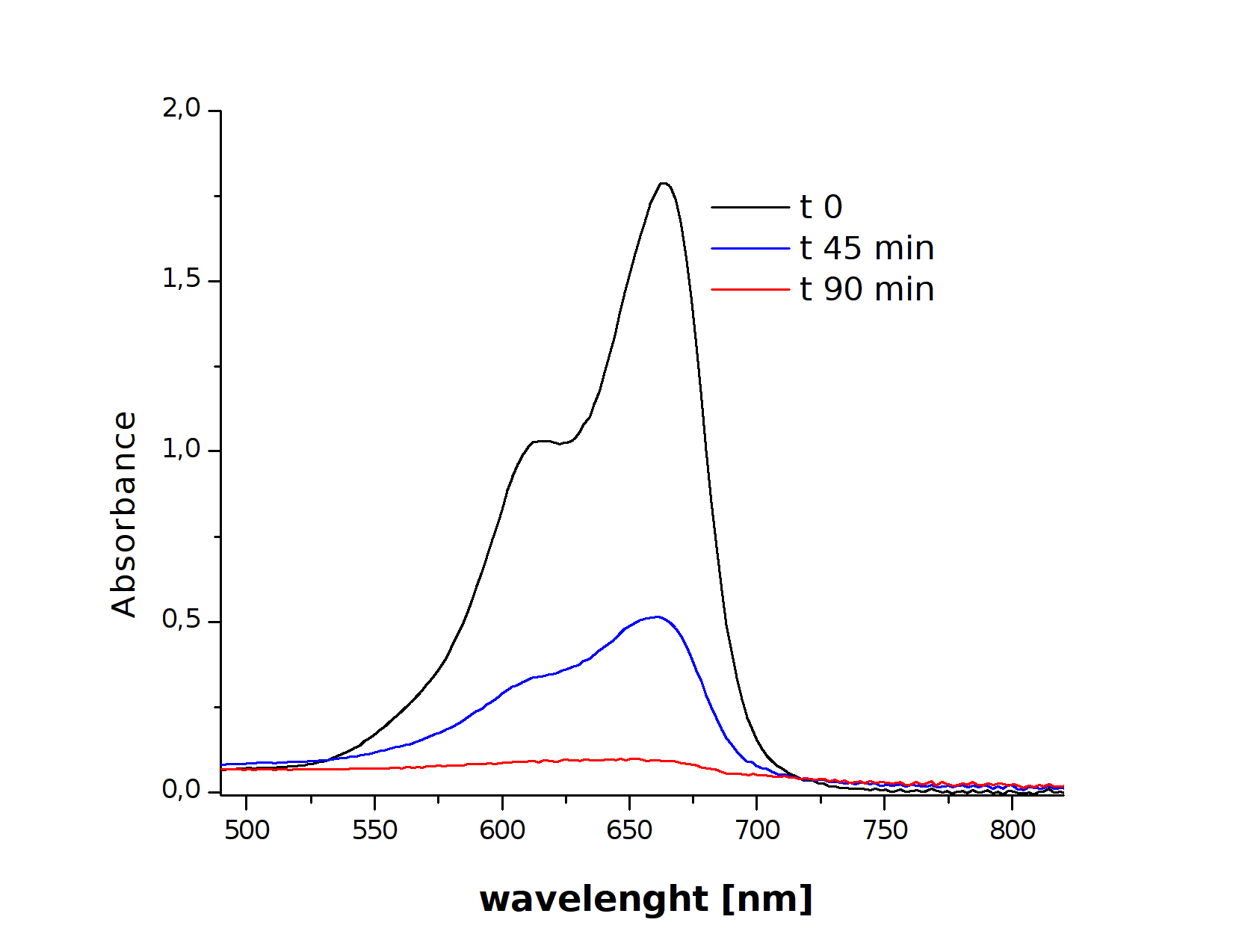


**Figure S1**: Example of UV visible spectra of MB solution after ultrasonic treatment at 858 kHz without ZnO particles


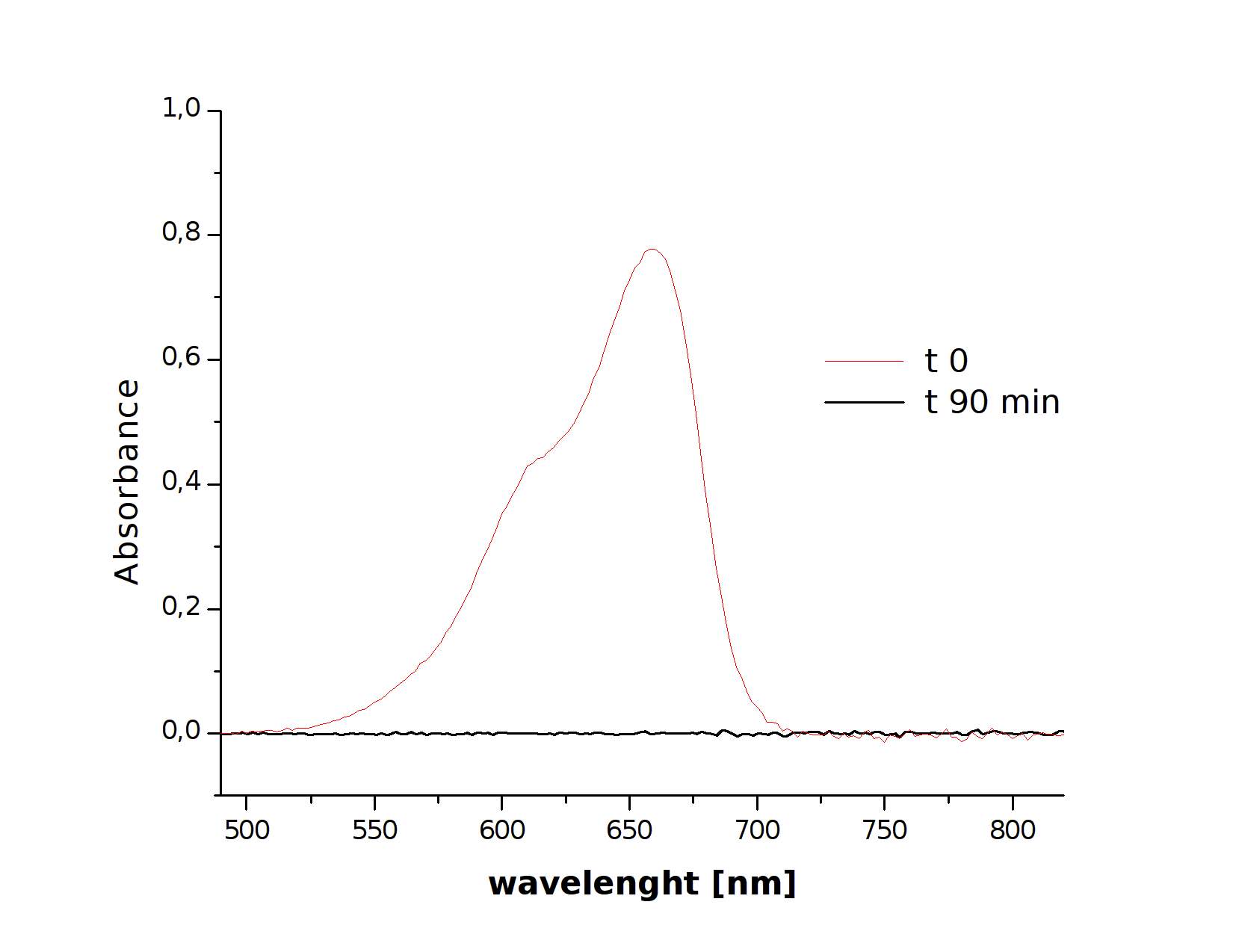


**Figure S2**: Example of UV visible spectra of MB solution after ultrasonic treatment at 858 kHz with ZnO nanorods


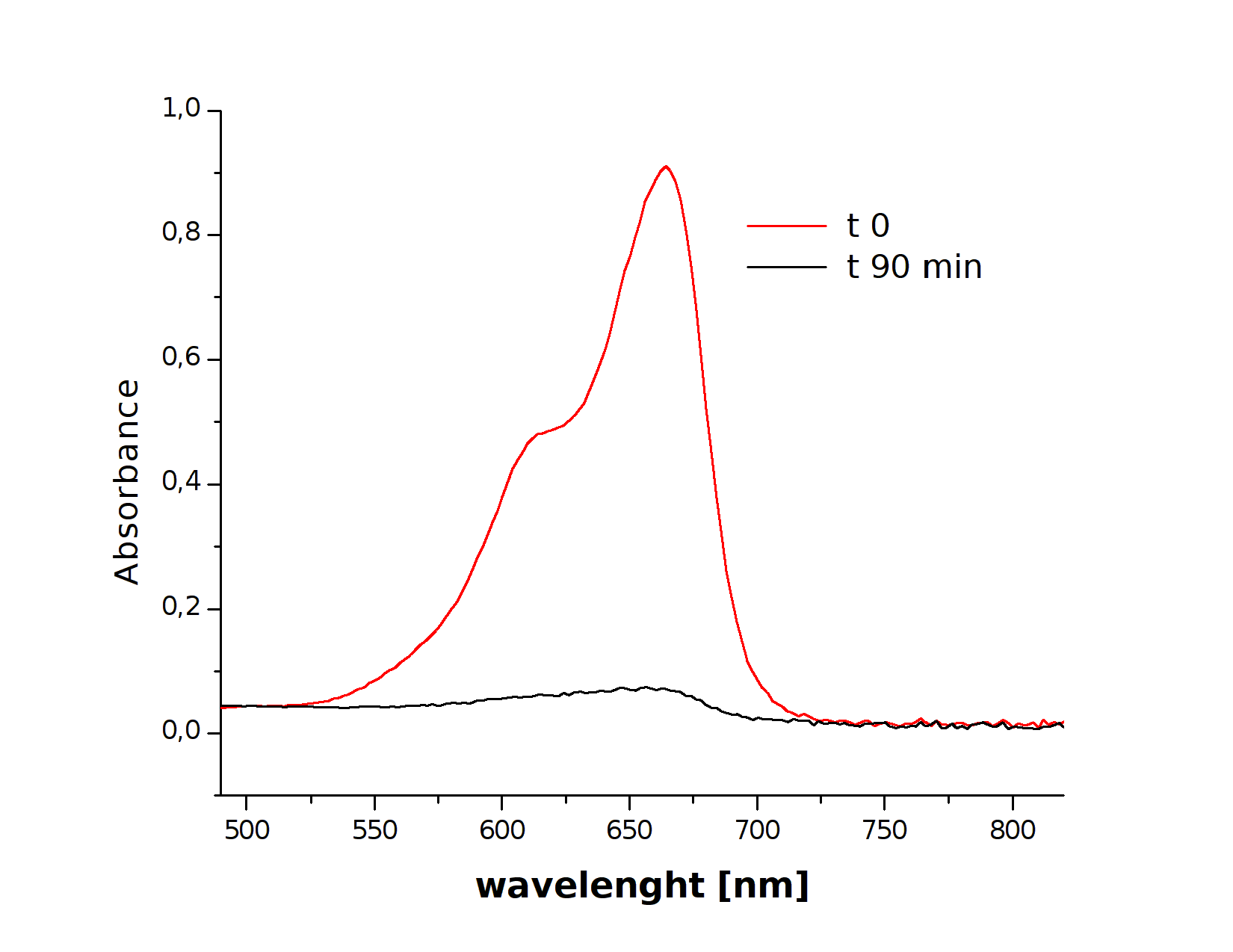


**Figure S3**: Example of UV visible spectra of MB solution after ultrasonic treatment at 20 kHz with ZnO nanorods
